# Development and Validation of a Nomogram Model for Predicting the Severity of Post‐Stroke Dysphagia Based on fNIRS‐Derived Brain Functional Connectivity Features

**DOI:** 10.1002/brb3.71791

**Published:** 2026-09-23

**Authors:** Bangqiang Hou, Qian Wen, Yiya Wang, Rong Zhang, Xiaojuan Chen, Yizheng Li, Yinxu Wang, Yulei Xie

**Affiliations:** ^1^ Department of Rehabilitation Medicine Affiliated Hospital of North Sichuan Medical College Nanchong China; ^2^ Department of Rehabilitation Medicine Nanchong Shunqing District People's Hospital Nanchong China; ^3^ Department of Rehabilitation Medicine Qinghai Provincial People's Hospital Xining China

**Keywords:** functional near‐infrared spectroscopy, nomogram, post‐stroke dysphagia, predictive model

## Abstract

**Objective:**

This study aimed to develop and validate a nomogram model based on resting‐state functional near‐infrared spectroscopy (fNIRS) data to predict the severity of post‐stroke dysphagia (PSD), providing a basis for individualized assessment and intervention for PSD.

**Methods:**

This multicenter retrospective study consecutively enrolled 178 PSD patients from two hospitals between March 2023 and April 2026. Patients were classified into mild (Standardized Swallowing Assessment [SSA] score < 26) and severe (SSA ≥ 26) PSD groups. Resting‐state fNIRS data were collected to calculate functional connectivity strength among 18 swallowing‐related brain regions, generating 513 candidate features. In the training set, least absolute shrinkage and selection operator (LASSO) regression with 10‐fold cross‐validation was first used for preliminary feature screening, followed by Bootstrap resampling (*B* = 200) for stability selection to identify final predictors. A multivariate logistic regression model was constructed based on the selected features, and a visualized nomogram was established accordingly. Model performance was comprehensively evaluated: discriminative ability via the area under the receiver operating characteristic curve (AUC), calibration via calibration curves, and clinical utility via decision curve analysis (DCA).

**Results:**

Three stable functional connectivity features were finally included in the model: LIPG_RIPG (interhemispheric connectivity of the inferior prefrontal gyrus), RDLPFC_RVAC (connectivity between right dorsolateral prefrontal cortex and right visual association cortex), and LFEF_LFEF (intraregional connectivity of left frontal eye field). The model demonstrated excellent discriminative performance, with AUCs of 0.928 (95% CI: 0.878–0.978) in the training set, 0.857 (95% CI: 0.727–0.986) in the internal validation set, and 0.881 (95% CI: 0.769–0.994) in the external validation set. Calibration curves showed high consistency between predicted risk and actual observation, and DCA confirmed the model yielded positive net clinical benefit across a wide threshold probability range (0.05–0.9). Sensitivity analyses further verified that the predictive value of the three fNIRS features was independent of conventional clinical variables and not affected by the choice of PSD severity cutoff.

**Conclusion:**

The nomogram model based on fNIRS functional connectivity can effectively predict PSD severity, with sound discrimination, calibration, and clinical translational potential. It provides clinicians with a non‐invasive, easy‐to‐use quantitative tool for early identification of high‐risk PSD patients and supports evidence‐based formulation of personalized rehabilitation strategies, promoting the transition of PSD management from subjective scale assessment to objective neurofunctional precision evaluation.

## Graphical abstract

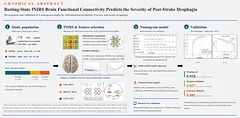

## 1 Introduction

Post‐stroke dysphagia (PSD) is a common and severe complication following stroke, with a prevalence ranging from 8.1% to 80%. Particularly during the acute phase, its incidence can be as high as 42% to 75% (Banda et al. 2022; Labeit et al. 2024). PSD not only severely impairs patients' nutritional intake and quality of life but is also directly associated with serious complications such as aspiration and aspiration pneumonia. Studies indicate that PSD can increase the risk of pneumonia in stroke patients by approximately fourfold and significantly elevate mortality rates. Additionally, it may lead to dehydration, electrolyte imbalances, and malnutrition, further exacerbating the disease burden on patients and increasing socio‐economic pressure on families and society (Banda et al. 2022). Therefore, within the stroke management system, early and accurate assessment of swallowing function is a critical step for identifying high‐risk patients, formulating individualized rehabilitation plans, and improving prognosis. Delayed assessment is often accompanied by an increased risk of pneumonia and prolonged hospital stays. Currently, commonly used clinical screening and assessment tools include the Standardized Swallowing Assessment (SSA) and the Water Swallowing Test (WST), among others. While these tools offer advantages such as operational simplicity and ease of bedside implementation, their assessment results largely depend on the operator's subjective judgment and clinical experience, lacking sufficient support from objective quantitative indicators (Banda et al. 2022). Several studies have constructed nomograms for early prediction and risk assessment across various clinical settings, incorporating different types of predictors, ranging from acoustic clinical features for lung cancer to routine clinical parameters for bacterial bloodstream infection. These models provide a methodological reference for integrating diverse features into a user friendly scoring system (Jiang et al. 2025; Lu, Sha, et al. 2025).

Functional near‐infrared spectroscopy (fNIRS) is a non‐invasive, portable brain functional imaging technology. Based on the neurovascular coupling mechanism, it detects concentration changes of oxygenated hemoglobin (HbO) and deoxygenated hemoglobin (HbR) in the cerebral cortex, thereby reflecting local brain area activation and brain network functional connectivity status in real‐time (Hou, Liu, et al. 2025; Ma et al. 2024). This technology offers relatively high temporal resolution, good motion tolerance, and lower cost and has demonstrated significant value in assessing motor and cognitive functions after stroke (H. Liu et al. 2022; Ma et al. 2024). Swallowing, as a complex neurobehavioral act, relies on the coordinated regulation of multiple brain regions, including the prefrontal cortex, primary motor cortex, sensory cortex, and supplementary motor area (Hou, Liu, et al. 2025; Hou, Xie, et al. 2025; Ma et al. 2024). Previous studies and preliminary research by our team have shown that the functional connectivity strength in corresponding brain regions is significantly reduced in PSD patients and is negatively correlated with SSA scores. This suggests that weakened brain network connectivity may be the neural basis for impaired swallowing function (Hou, Liu, et al. 2025; Hou, Xie, et al. 2025; Wang, Ma et al. 2025). Furthermore, fNIRS can capture abnormal patterns of brain region activation during swallowing tasks in patients, such as compensatory enhancement in the unaffected hemisphere or insufficient activation in the affected hemisphere, further revealing the association between brain functional reorganization and dysphagia (Ma et al. 2024; D. Zhao et al. 2025). This provides a theoretical basis for the application of fNIRS in dysphagia assessment and intervention target localization.

Therefore, this study aimed to develop and validate a nomogram model by leveraging fNIRS‐derived functional connectivity features to predict PSD severity. This model is intended to provide an objective and quantifiable tool to supplement current clinical assessments.

## 2 Materials and Methods

### 2.1 Study Subjects

The study protocol was reviewed and approved by the Medical Ethics Committee of the Affiliated Hospital of North Sichuan Medical College (Approval No. 2026ER205‐1) and the Medical Ethics Committee of Sichuan Gem Flower Hospital (Approval No. SCBSH‐EC‐26‐23). The study was conducted in strict accordance with the Declaration of Helsinki and relevant ethical guidelines. Given that this was a retrospective study, the ethics committees granted a waiver of informed consent. Prior to data collation and analysis, all patient information underwent de‐identification to ensure full protection of privacy.

The subjects were recruited from outpatients and inpatients diagnosed with stroke at the Affiliated Hospital of North Sichuan Medical College (from March 2023 to November 2025) and Sichuan Gem Flower Hospital (from May 2024 to April 2026). Case screening was strictly performed based on the following inclusion and exclusion criteria:

Inclusion Criteria: (1) Diagnosis of unilateral stroke confirmed by computed tomography (CT) or magnetic resonance imaging (MRI) (Song et al. 2024); (2) Age ≥ 18 years; (3) Presence of dysphagia, defined as a WST grade ≥ 3, and/or confirmed penetration or aspiration by Fiberoptic Endoscopic Evaluation of Swallowing (FEES) (Jamil et al. 2025).

Exclusion Criteria: (1) Dysphagia caused by other neurological disorders (e.g., Parkinson's disease, Alzheimer's disease, traumatic brain injury, cerebellar or brainstem stroke, head and neck tumors); (2) Presence of severe aphasia, cognitive impairment, or consciousness disturbance preventing cooperation with required examinations; (3) Absence of dysphagia; (4) Missing or poor‐quality SSA or fNIRS data (Non‐unilateral stroke and dysphagia due to other conditions were also excluded.)

Following screening according to the aforementioned criteria, a total of 140 PSD patients from the Affiliated Hospital of North Sichuan Medical College and 38 PSD patients from Sichuan Gem Flower Hospital were ultimately enrolled. Patients were categorized into two groups based on the SSA score: those with an SSA score < 26 were assigned to the mild PSD group (MPSDG), while those with an SSA score ≥ 26 were assigned to the severe PSD group (SPSDG). In this study, the presence or absence of severe swallowing dysfunction served as the binary outcome variable (D'Netto et al. 2023; González‐Fernández et al. 2013; Wang ，Chen, et al. 2025). Using a random sampling method, the 140 PSD patients from the Affiliated Hospital of North Sichuan Medical College were divided into a training set (*n* = 100) for model construction and an internal validation set (*n* = 40) for model verification, at a ratio of 7:3. Patients from Sichuan Gem Flower Hospital constituted the external validation set (*n* = 38).

### 2.2 Data Acquisition

(1) Basic patient information was extracted from the electronic medical record system. This included age, sex, stroke type, stroke hemisphere, hypertension, heart disease, diabetes, smoking status, alcohol consumption, and the SSA score upon admission.

(2) A 63‐channel fNIRS system (NirSmart, Huichuang, Danyang, China) was used to collect 5‐min resting‐state data from PSD patients. During acquisition, subjects were instructed to remain awake and relaxed and to minimize head movements and swallowing actions to reduce artifacts. Following data acquisition, the preprocess module of NirSpark software was utilized for raw data preprocessing. Initially, the signal standard deviation (SD) threshold was set to 6 and the peak threshold to 0.5. Spline interpolation was applied to identify and remove motion artifacts, thereby ensuring data accuracy. Subsequently, a band‐pass filter (0.01–0.1 Hz) was employed to eliminate physiological noise—including cardiac cycles, respiration, and Mayer waves—to extract neural activity signals during the resting state. During signal processing, the pathlength differential factor was configured within the range of −6 to 6. Based on the modified Beer‐Lambert law (MBLL), real‐time signal changes in oxyhemoglobin (HbO), deoxyhemoglobin (HbR), and total hemoglobin (HbT) were calculated for patients during the resting task. Considering the performance differences in signal‐to‐noise ratio, sensitivity to neural activity, and statistical power, this study determined that HbO, HbR, and HbT should be included simultaneously in the analysis (Hou, Xie, et al. 2025; Lin et al. 2023; Shah and Seghouane 2014; Tachtsidis and Scholkmann 2016).

Based on this, 18 regions of interest (ROIs) were defined according to swallowing‐related brain network research. These ROIs covered the left/right primary motor cortex (L/RPMC), left/right primary somatosensory cortex (L/RPSC), left/right pre‐motor and supplementary motor cortex (L/RpSMC), left/right supramarginal gyrus (L/RSMG), left/right dorsolateral prefrontal cortex (L/RDLPFC), left/right frontal eye fields (L/RFEF), left/right frontopolar area (L/RFPA), left/right inferior prefrontal gyrus (L/RIPG), and left/right visual association cortex (L/RVAC) (Cheng et al. 2022; Kang et al. 2024). The channels and source‐detector pairs corresponding to the ROIs are listed in Table 1.

**TABLE 1:** The channels and source‐detector pairs corresponding to the ROIs.

ROIs	Channels	S + D
**LpSMC**	27, 28, 36, 37, 38, 40, 41, 54, 57	S11, S14, S15, S20, S21, D15, D18, D21, D22
**RpSMC**	30, 31, 32, 33, 46, 47, 49, 50, 51	S12, S17, S18, S19, D14, D17, D19, D20
**LPSC**	13, 14, 15, 52, 53, 55, 56,	S6, S2, 12, S21, D5, D11, D21, D22
**RPSC**	1, 2, 16, 17, 44, 45, 48	S1, S7, S17, S18, D1, D12, D19
**LPMC**	27, 28, 38, 54, 57	S11, S14, S20, S21, D15, D21, D22
**RPMC**	30, 32, 46, 47, 49	S12, S17, S18, D12, D17, D19, D20
**LIPG**	11, 12	S5, D4, D10
**RIPG**	4, 3	S2, D2, D7
**LFPA**	8, 9, 10, 21, 23, 24, 25	S4, S9, S10, D3, D4, D9
**RFPA**	5, 6, 7, 18, 20, 21, 22	S3, S8, S9, D2, D3, D8
**LDLPFC**	24, 26, 29	S10, S11, D4, D10, D23
**RDLPFC**	18, 19, 34	S8, S13, D2, D7, D17
**LSMG**	13, 15, 52	S6, S20, D5, D22
**RSMG**	1, 2, 48	S1, S18, D1, D12
**LFEF**	29, 37, 39	S11, S14, D18, D23
**RFEF**	34, 35, 51	S13, S19, D17, D20
**LVAC**	60, 61, 62, 63	S23, S24, D16, D24
**RVAC**	42, 43, 58, 59	S16, S22, D6, D13

Abbreviations: D, detector; L/RDLPFC, left/right dorsolateral prefrontal cortex; L/RFEF, left/right frontal eye fields; L/RFPA, left/right frontopolar area; L/RIPG, left/right inferior prefrontal gyrus; L/RPMC, left/right primary motor cortex; L/RPSC, left/right primary somatosensory cortex; L/RpSMC, left/right pre‐motor and supplementary motor cortex; L/RSMG, left/right supramarginal gyrus; L/RVAC, left/right visual association cortex; ROIs, regions of interest; S, source.

**FIGURE 1:**
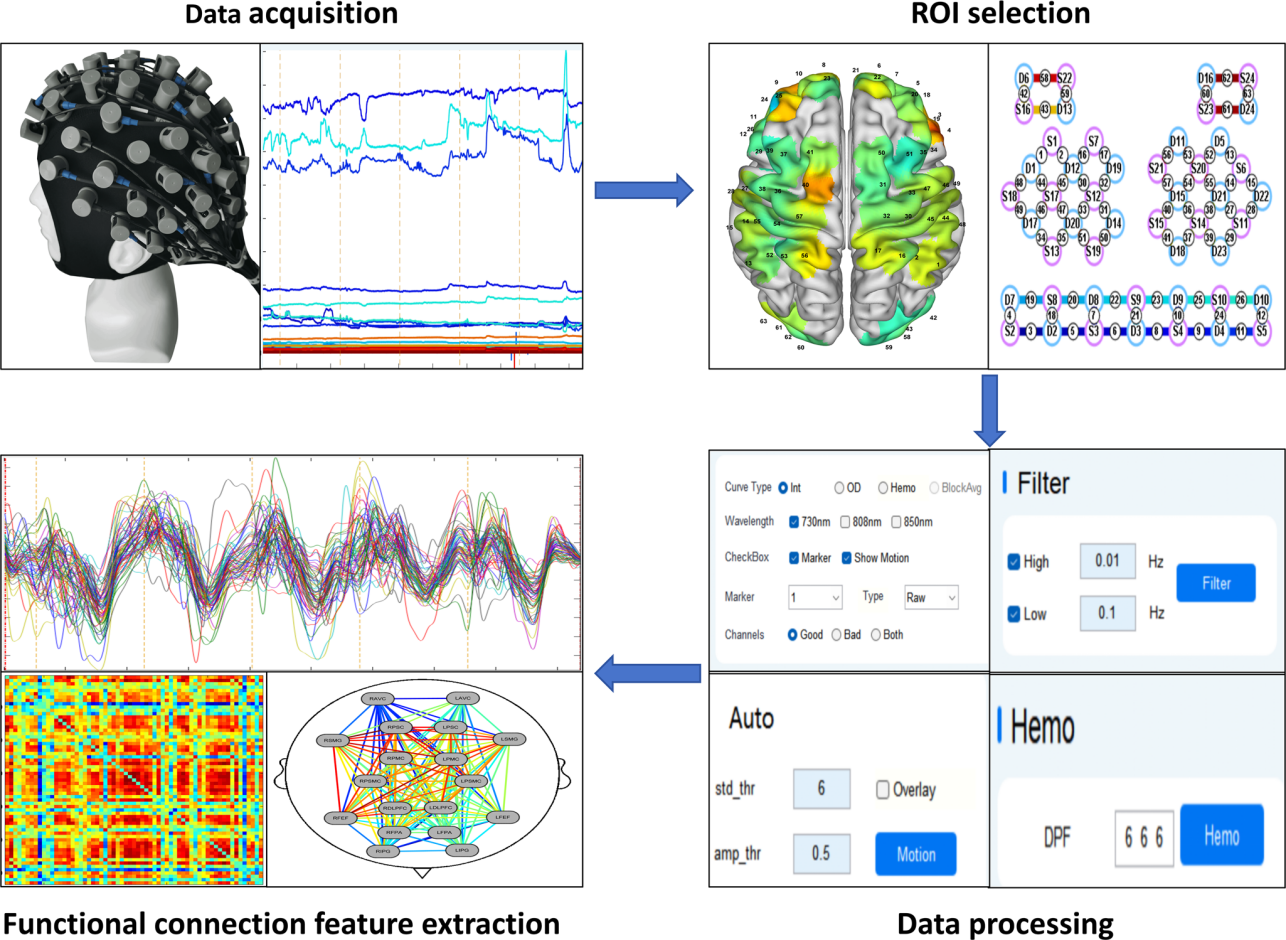
fNIRS data acquisition and analysis pipeline.

Functional connectivity features were then extracted within each ROI (ROI‐self) and between each pair of ROIs (ROI‐ROI) using the Network module of the NirSpark software. By calculating the Pearson correlation coefficient of the time series and applying Fisher's z‐transformation, a total of 513 functional connectivity features were obtained, which served as candidate predictor variables for subsequent analysis. The fNIRS data acquisition and analysis pipeline is illustrated in Figure 1.

### 2.3 Statistical Analysis

Statistical analyses were performed using R software (version 4.6.0). For descriptive statistics of demographic and clinical characteristics, continuous variables are presented as mean ± (SD), while categorical variables are expressed as frequencies (percentages). Comparisons of continuous variables between groups were conducted using the independent samples *t*‐test, Mann–Whitney *U*‐test, or Kruskal–Wallis test, as appropriate. Categorical variables were compared using the chi‐square (*χ* ^2^) test or Fisher's exact test. All statistical tests were two‐sided, and a *p*‐value < 0.05 was considered statistically significant.

Using stratified random sampling based on SSA status, 140 patients with PSD were randomly allocated to the training and internal validation sets at a 7:3 ratio. A fixed random seed was specified to ensure reproducibility. Candidate features were preprocessed exclusively within the training set. Features with near‑zero variance were first excluded to eliminate low‐information variables. All remaining features were then standardized using *Z*‐score normalization derived from the training set, and identical transformation parameters were applied to the internal validation set. Feature selection was performed using LASSO logistic regression with 10‐fold cross‑validation to identify the optimal penalty parameter *λ*. Variables with non‐zero coefficients at the *λ*.1se value were retained as the candidate feature set. To further assess selection stability, bootstrap resampling (*B* = 200) was implemented; the LASSO procedure was repeated on each resampled dataset, and the selection frequency of each candidate feature was calculated. Features selected in ≥ 60% of bootstrap iterations were defined as stable predictors and entered into the final model. A multivariable logistic regression model was subsequently fitted using these stable features. Regression coefficients, odds ratios (ORs), and corresponding 95% confidence intervals (CIs) were estimated and reported. Finally, a nomogram was constructed based on the final model to enable individualized risk prediction for severe PSD.

Model discriminative ability was evaluated using the receiver operating characteristic (ROC) curve. The area under the curve (AUC) was calculated in the training set, internal validation set, and external validation set, and the 95% CI of the AUC was obtained using the DeLong method. When necessary, the DeLong test was used to compare the difference in AUC between the multivariable model and the univariable model.

To further evaluate the stability and generalization ability of the final model, this study performed repeated fivefold cross‐validation within the training set. This process was repeated five times, yielding a total of 25 model evaluation results. For each fold, a logistic regression model containing the three selected features was fitted on the training subset and evaluated on the corresponding validation subset. The AUC for each iteration was recorded, and the mean, SD, median, and range of the AUC values were calculated. Additionally, the sensitivity and specificity at the default probability threshold of 0.5 were summarized.

Meanwhile, we embedded the synthetic minority over‐sampling technique (SMOTE) and the random over‐sampling examples (ROSE) algorithms within the repeated fivefold cross‑validation loop to rebalance the training set (from 1:3 to 1:1) and compared their performance with that of the original imbalanced primary model in order to investigate the potential classification bias arising from the 1:3 class imbalance.

Model calibration was assessed in the training set using bootstrap internal validation (*B* = 1000) to generate bias‐corrected calibration curves and calculate the mean absolute error (MAE). In the internal validation set and external validation set, the grouping method was used to calculate the observed event rate and mean predicted probability, calibration points were plotted, and the MAE was also reported.

Clinical benefit was evaluated using decision curve analysis (DCA), and the net benefit at different threshold probabilities was calculated and compared across the training set, internal validation set, and external validation set. All statistical tests were two‐sided, and a *p* value < 0.05 was considered statistically significant.

All modeling procedures were performed in R. Specific R packages utilized included “caret” (for data partitioning and preprocessing), “glmnet” (for LASSO feature selection), “rms” (for logistic regression modeling, nomogram construction, and model calibration), “pROC” (for ROC curve analysis and AUC calculation), and “rmda” (for DCA).

## 3 Results

### 3.1 General Characteristics

**FIGURE 2:**
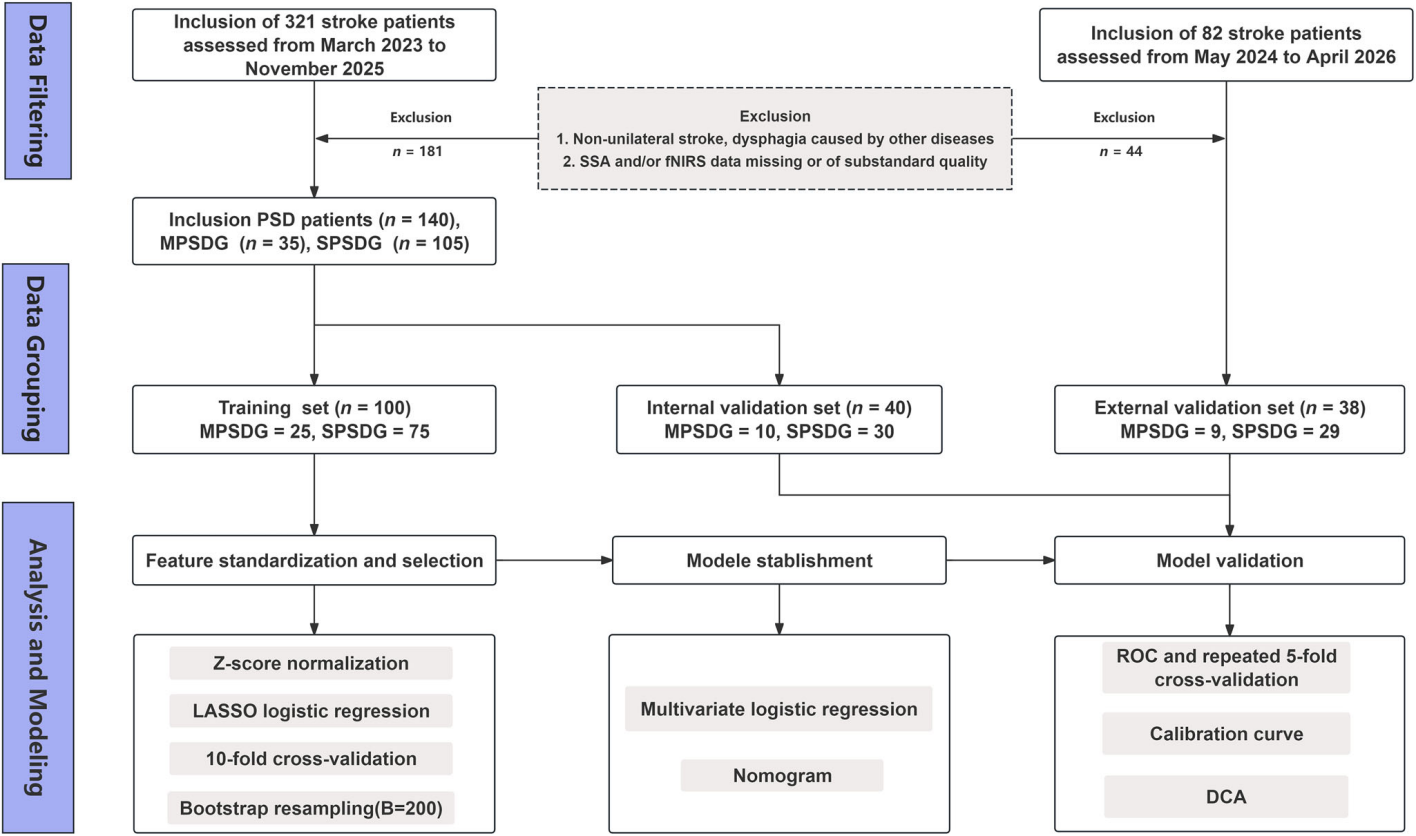
Flow diagram. DCA, decision curve analysis; MPSDG, mild PSD group; PSD, post‐stroke dysphagia; ROC, receiver operating characteristic; SPSDG, severe PSD group.

A total of 178 subjects were enrolled in this study, comprising 140 patients from the Affiliated Hospital of North Sichuan Medical College (allocated to the training set [*n* = 100] and internal validation set [*n* = 40]) and 38 patients from Sichuan Gem Flower Hospital (external validation set). Baseline demographic and clinical characteristics are summarized in Table 2 and Table 3; Table S1, with the screening flow diagram detailed in Figure 2.

**TABLE 2:** Baseline characteristics of the 140 PSD patients.

Variables	Total (*n* = 140)	MPSDG (*n* = 35)	SPSDG (*n* = 105)	Statistics	*p*
Age, (years), (median, IQR)	66.00 (58.00,74.75)	66.00 (55.50, 73.00)	67.00 (59.00, 75.00)	−1.119^a^	0.264
Sex, *n* (%)				0.041^b^	0.837
Male	90 (64.29)	22 (62.86)	68 (64.76)		
Female	50 (35.71)	13 (37.14)	37 (35.24)		
Stroke types, *n* (%)				1.574^b^	0.210
Ischemic	114 (81.4)	26 (74.3)	88 (83.8)		
Hemorrhagic	26 (18.6)	9 (25.7)	17 (16.2)		
Site of stroke, *n* (%)				0.010^b^	0.922
Left	61 (43.6)	15 (42.9)	46 (43.8)		
Right	79 (56.4)	20 (57.1)	59 (56.2)		
Hypertension, *n* (%)				0.563^b^	0.453
No	41 (29.3)	12 (34.3)	29 (27.6)		
Yes	99 (70.7)	23 (65.7)	76 (72.4)		
Heart disease, *n* (%)				0.057^b^	0.812
No	110 (79.6)	28 (80.0)	82 (78.1)		
Yes	30 (21.4)	7 (20.0)	23 (21.9)		
Diabetes, *n* (%)				0.163^b^	0.686
No	88 (62.9)	21 (60.0)	67 (63.8)		
Yes	52 (37.1)	14 (40.0)	38 (36.2)		
Smoking, *n* (%)				0.353^b^	0.552
No	82 (58.6)	22 (62.9)	60 (57.1)		
Yes	58 (41.4)	13 (37.1)	45 (42.9)		
Drinking, *n* (%)				0.635^b^	0.426
No	84 (60)	23 (65.7)	61 (58.1)		
Yes	56 (40)	12 (34.3)	44 (41.9)		
SSA (median, IQR)	29.00 (26.00, 33.00)	23.00 (22.00, 24.00)	31.00 (28.00, 34.00)		

Abbreviations: MPSDG, mild PSD group; PSD, post‐stroke dysphagia; SPSDG, severe PSD group; SSA, Standardized Swallowing Assessment.

^a^ Mann–Whitney U test;

^b^ Chi‐square (χ²) test

**TABLE 3:** Baseline characteristics of PSD patients in the training, internal validation, and external validation sets.

Variables	Training (*n* = 100)	Internal validation (*n* = 40)	External validation (*n* = 38)	Statistics	*p*
Severity, *n* (%)				0.028^b^	0.986
MPSDG	25 (25)	10 (25)	9 (23.7)		
SPSDG	75 (75)	30 (75)	29 (76.3)		
Age, (years), (median, IQR)	66.50 (57.00, 74.75)	65.50 (59.25, 74.75)	68.00 (59.75, 74.00)	0.252^a^	0.882
Sex, *n* (%)				0.288^b^	0.866
Male	59 (59.0)	23 (57.5)	24(63.2)		
Female	41 (41.0)	17 (42.5)	14(36.8)		
Stroke causes, *n* (%)				3.865^b^	0.145
Ischemic	84 (84.0)	30 (75.0)	28 (73.7)		
Hemorrhagic	16 (16.0)	10 (25.0)	10(26.3)		
Stroke site, *n* (%)				1.105^b^	0.576
Left	44 (44.0)	17 (42.5)	13 (34.2)		
Right	56 (56.0)	23 (57.5)	25 (65.8)		
Hypertension, *n* (%)				0.161^b^	0.923
No	30 (30.0)	11 (27.5)	12 (31.6)		
Yes	70 (70.0)	29 (72.5)	26 (68.4)		
Heart disease, *n* (%)				0.906^b^	0.636
No	77 (77.0)	33 (82.5)	28 (73.7)		
Yes	23 (23.0)	7 (17.5)	10 (26.3)		
Diabetes, *n* (%)				0.505^b^	0.777
No	64 (64.0)	24 (60.0)	22 (57.9)		
Yes	36 (36.0)	16 (40.0)	16 (42.1)		
Smoking, *n* (%)				0.981^b^	0.612
No	61 (61.0)	21 (52.5)	21 (55.3)		
Yes	39 (39.0)	19 (47.5)	17 (44.7)		
Drinking, *n* (%)				1.317^b^	0.518
No	63 (63.0)	21 (52.5)	23 (60.5)		
Yes	37 (37.0)	19 (47.5)	15 (39.5)		
SSA (median, IQR)	29.00 (26.00, 33.00)	29.00 (26, 32.75)	28.00 (25.75, 32.00)	1.131^a^	0.568

Abbreviations: MPSDG, mild PSD group; PSD, post‐stroke dysphagia; SPSDG, severe PSD group; SSA, Standardized Swallowing Assessment.

^a^ Kruskal–Wallis test;

^b^ Chi‐square (χ²) test.

Intergroup comparisons revealed that the three groups were well‐balanced regarding age, sex, stroke type, affected hemisphere, and medical history (hypertension, heart disease, diabetes, smoking, and alcohol consumption), with all *p* > 0.05. These results demonstrate that the randomization was effective and the groups were comparable. Detailed statistical analyses are presented in Table 3.

### 3.2 Feature Standardization and Selection

To eliminate the influence of scale differences among functional connectivity features on model stability, all continuous features were standardized using *Z*‐score normalization before model construction, transforming them into a distribution with a mean of 0 and a SD of 1.

During the feature selection stage, preliminary screening was first performed in the training set using LASSO logistic regression with 10‐fold cross‐validation. Features with non‐zero coefficients corresponding to *λ*.1se were selected as the candidate variable set. To evaluate the robustness of the feature selection, stability selection was further conducted using bootstrap resampling. Stable features that were selected with high frequency across repeated resampling iterations were retained.

**FIGURE 3:**
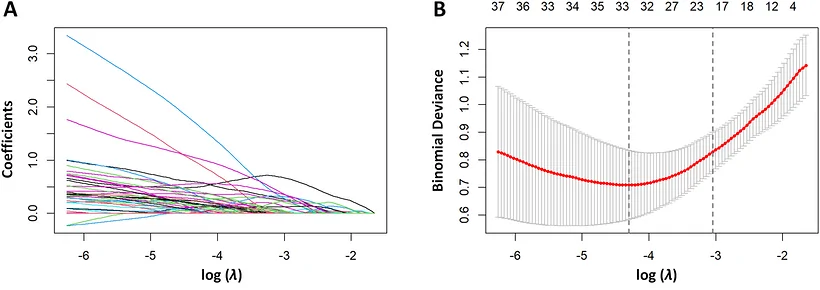
LASSO regression analysis based on 10‐fold cross‐validation for predicting PSD. (A) LASSO coefficient path plot: This plot illustrates the trajectories of regression coefficients for all feature variables as the regularization parameter *λ* (presented as Log Lambda) varies. Each curve represents a feature, with the vertical axis showing the coefficient value and the horizontal axis showing Log Lambda. As the *λ* value increases (moving rightward), the penalty strength intensifies, causing the coefficients of some features to shrink gradually toward zero, thereby achieving automatic feature selection. Features with non‐zero coefficients were ultimately retained as predictors. (B) LASSO cross‐validation deviance plot: This plot displays the relationship between model deviance (binomial deviance) and the *λ* value via 10‐fold cross‐validation. The red curve represents the mean deviance, and the gray shaded area indicates the standard error range of the deviance. The two vertical dashed lines correspond to the *λ* at which the deviance is minimized (*λ*.min) and the more parsimonious *λ* within one standard error (*λ*.1se). In this study, *λ*.1se was selected as the optimal parameter to balance model complexity and predictive stability.

Figure 3 illustrate the feature selection process based on LASSO regression. Figure 3A presents the LASSO coefficient path plot, where the vertical axis represents the regression coefficients and the horizontal axis is the logarithm of the penalty parameter *λ* [log(*λ*)], showing the gradual shrinkage of each feature's coefficient as the penalty strength increases. Figure 3B displays the 10‐fold cross‐validation deviance curve, where the lowest point on the curve corresponds to the *λ* value associated with the minimum prediction error.

**FIGURE 4:**
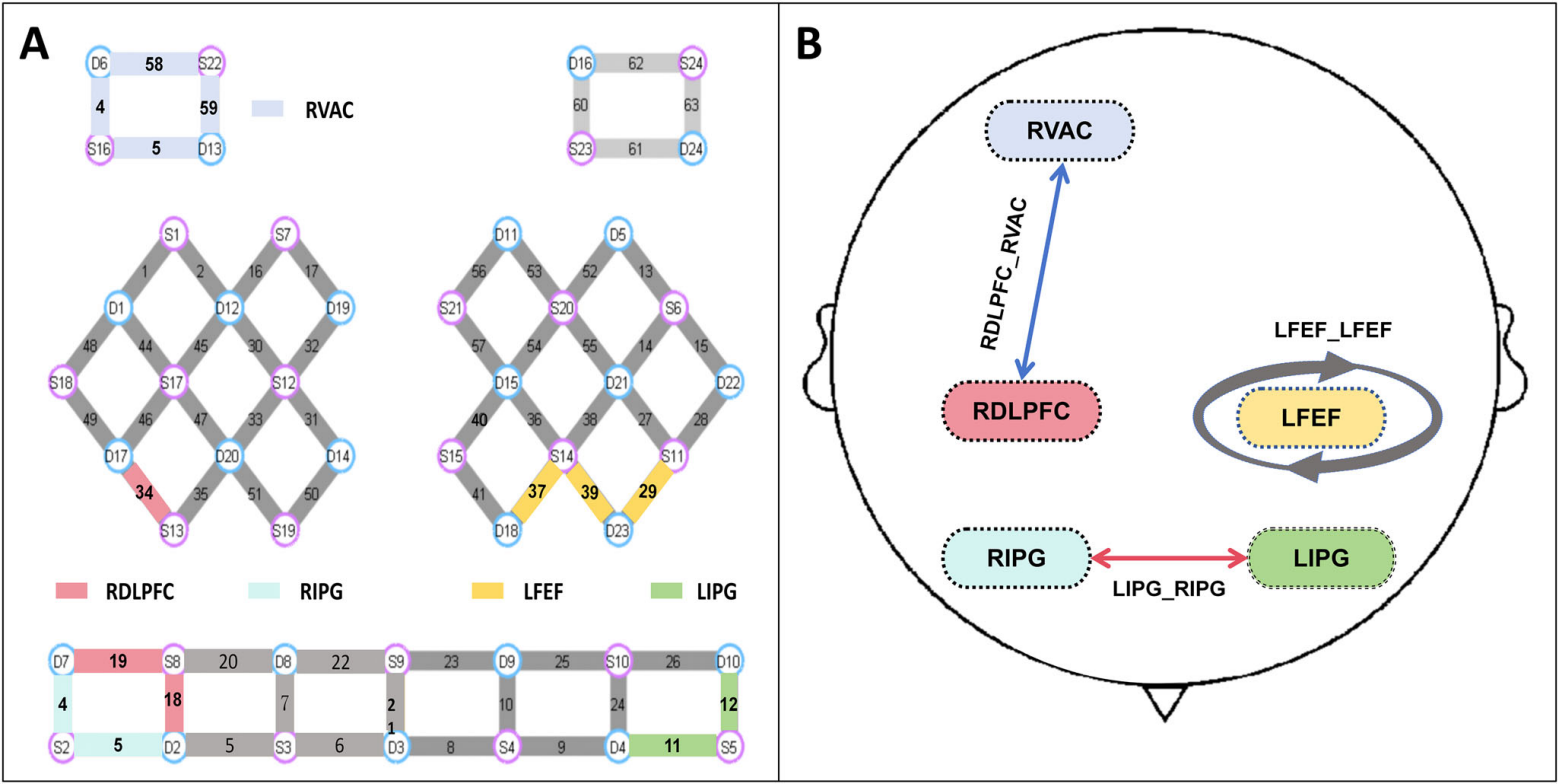
Schematic diagram of brain network based on functional connectivity features. L/RIPG, left/right inferior prefrontal gyrus; LFEF, left frontal eye fields; LFEF_LFEF, intra‐regional functional connectivity within the LFEF; LIPG_RIPG, functional connectivity between the LIPG and RIPG; RDLPFC, right dorsolateral prefrontal cortex; left frontal eye fields; RDLPFC_RVAC, functional connectivity between the RDLPFC and RVAC; RVAC, right visual association cortex.

Based on the feature selection results, a multivariate logistic regression model was constructed to predict severe PSD. The key predictors identified were functional connectivity between the LIPG and the RIPG (LIPG_RIPG), functional connectivity between the RDLPFC and the RVAC (RDLPFC_RVAC), and intra‐regional functional connectivity within the LFEF (LFEF_LFEF). Notably, these features represent functional connectivity strength indicators under the HbO condition and exhibited robust discriminative ability in both univariate analysis and cross‐validation (Table 4). A schematic diagram of the brain network based on functional connectivity features is shown in Figure 4.

**TABLE 4:** Final model coefficients.

Feature	B	SE	OR	95% CI	*p* value
LIPG_RIPG	2.11	0.69	8.21	2.12–31.77	< 0.01
RDLPFC_RVAC	2.29	0.75	9.91	2.30–42.75	< 0.01
LFEF_LFEF	2.10	0.66	8.17	2.26–29.50	< 0.01

Abbreviations: B, regression coefficient; CI, confidence interval; L/RIPG, left/right inferior prefrontal gyrus; LFEF, left frontal eye fields; LFEF_LFEF, intra‐regional functional connectivity within the LFEF; LIPG_RIPG, functional connectivity between the LIPG and RIPG; OR, odds ratio; RDLPFC, right dorsolateral prefrontal cortex; RDLPFC_RVAC, functional connectivity between the RDLPFC and RVAC; RVAC, right visual association cortex; SE, standard error.

### 3.3 Model Construction

**FIGURE 5:**
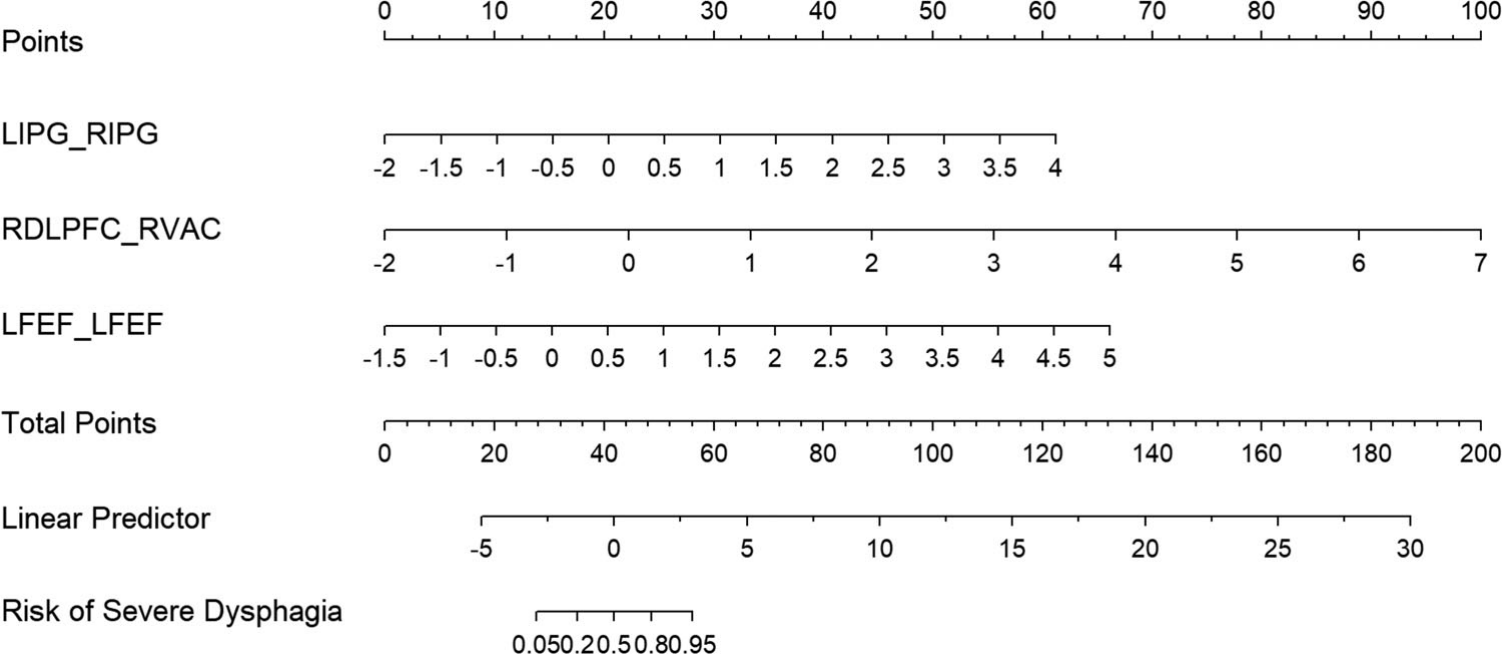
Nomogram for predicting the risk of severe PSD. Points: Reflect the contribution of each variable to the prediction; each variable value corresponds to a specific score. Total Points: The sum of scores for all variables; a higher total score indicates a greater risk of dysfunction. Predictor Variable Scales: Include three functional connectivity strength indicators: LIPG_RIPG (range: −2 to 4), RDLPFC_RVAC (range: −2 to 7), and LFEF_LFEF (range: −1.5 to 5). Linear Predictor: Converts the total score to a linear predictor value, serving as an intermediate step for probability calculation. Predicted Probability: Displays the predicted probability of an individual developing severe PSD, derived by transforming the linear predictor value via the logistic function. L/RIPG, left/right inferior prefrontal gyrus; LFEF, left frontal eye fields; LFEF_LFEF, intra‐regional functional connectivity within the LFEF; LIPG_RIPG, functional connectivity between the LIPG and RIPG; RDLPFC, right dorsolateral prefrontal cortex; left frontal eye fields; RDLPFC_RVAC, functional connectivity between the RDLPFC and RVAC; RVAC, right visual association cortex.

Based on the selected features, a multivariate logistic regression prediction model was constructed in the training set. The final model incorporated three variables with independent predictive value: LIPG_RIPG, RDLPFC_RVAC, and LFEF_LFEF. Using the regression coefficients of these variables, we further developed a nomogram for the individualized prediction of PSD severity probability, as detailed in Figure 5. The nomogram provides an intuitive visualization tool for individualized risk estimation, assisting clinicians in rapidly assessing a patient's risk of swallowing function recovery and providing a reference for formulating personalized rehabilitation plans. The variables included in the nomogram are described as follows:

1. Points and Total Points: The nomogram developed in this study includes three predictor variables, all of which are indicators of brain functional connectivity strength under HbO monitoring. The meaning, value range, and corresponding scoring rules in the nomogram for each variable are as follows: LIPG_RIPG: Represents the functional connectivity strength between LIPG and RIPG, with a value range of −2 to 4. For example, a patient value of 0 for this variable corresponds to 20 points on the nomogram. RDLPFC_RVAC: Represents the functional connectivity strength between RDLPFC and RVAC, with a value range of −2 to 7. For example, a patient value of 0 corresponds to 23 points. LFEF_LFEF: Represents the functional connectivity strength between LFEF and LFEF, with a value range of −1.5 to 5. For example, a patient value of 0 corresponds to 15 points. The individual scores for the above variables are summed to obtain the Total Points, which range from 0 to 200 points. A higher total score indicates a greater probability of the individual being at risk for severe PSD. For instance, the total score for the example patient described above would be 20 + 23 + 15 = 58 points.
2. Linear Predictor: This metric, ranging from −5 to 30, is a composite score obtained by the weighted linear combination of multiple predictor variables in the logistic regression model. For each patient, the values of all variables are weighted by their respective logistic regression coefficients and summed to produce a linear predictor value. This value is then mapped to the final risk probability via the sigmoid function. On the nomogram, clinicians can sum the points corresponding to each variable, locate this sum on the “Total Points” axis, and project it downward to the Linear Predictor scale to read the corresponding linear predictor value. This process visually demonstrates how multivariate information is integrated into a single risk assessment indicator. For example, the patient with a total score of 58 points, mentioned earlier, would have a corresponding linear predictor value of approximately 3.
3. Predicted Probability: This metric represents the predicted probability of an individual developing severe PSD, with a value range of 0.05 to 0.95. On the nomogram, the linear predictor value for each patient is converted to its corresponding occurrence probability via the logistic function. For example, if a patient has a linear predictor value of 3, the corresponding predicted probability scale is approximately 0.95 (i.e., 95%), indicating that the patient belongs to a high‐risk group. Clinicians can utilize such quantifiable risk assessments to formulate more targeted rehabilitation plans and follow‐up strategies for patients.

### 3.4 Predictive Model Performance and Validation

**FIGURE 6:**
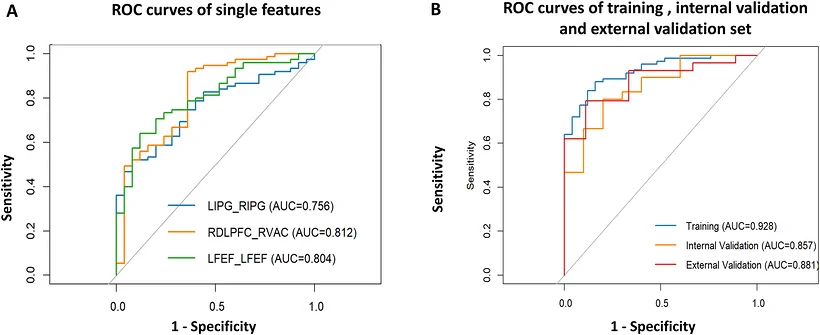
ROC curves for nomogram. L/RIPG, left/right inferior prefrontal gyrus; LFEF, left frontal eye fields; LFEF_LFEF, intra‐regional functional connectivity within the LFEF; LIPG_RIPG, functional connectivity between the LIPG and RIPG; RDLPFC, right dorsolateral prefrontal cortex; left frontal eye fields; RDLPFC_RVAC, functional connectivity between the RDLPFC and RVAC; RVAC, right visual association cortex.

To evaluate the discriminative ability, calibration, and clinical utility of the prediction model, we employed the AUC, calibration curves, and DCA for comprehensive validation. The results showed that the model exhibited good discriminative ability in the training set, internal validation set, and external validation set, with AUCs of 0.928 (95% CI: 0.878–0.978) in the training set, 0.857 (95% CI: 0.727–0.986) in the internal validation set, and 0.881 (95% CI: 0.769–0.994) in the external validation set, all higher than those of individual variables (Figure 6A,B).

In addition, a repeated fivefold cross‐validation with 25 iterations was performed within the training cohort. The cross‐validated mean AUC remained highly stable at 0.906 (SD = 0.070), confirming strong generalization. While the model exhibited an expected alignment with the real‐world sample distribution, showing a high sensitivity of 0.917 (SD = 0.065) and a lower specificity of 0.632 (SD = 0.214), the mathematically adjusted balanced accuracy achieved 0.775 (SD = 0.110), and the F1‐Score for the severe category was 0.899 (SD = 0.046). The validation results are provided in Figure S1 and S2.

**FIGURE 7:**
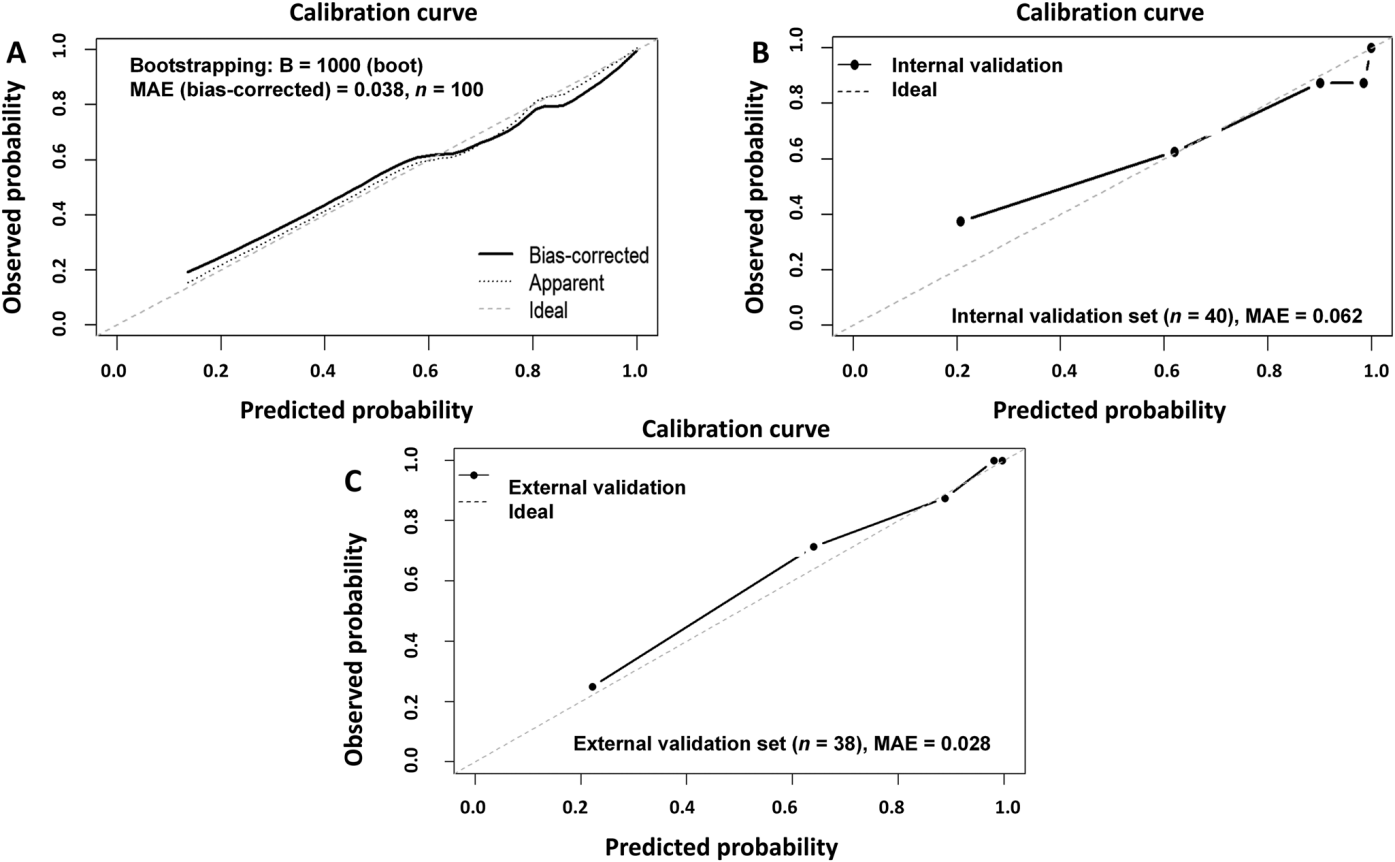
Calibration curves for the training, internal validation, and external validation set. MAE, mean absolute error.

Furthermore, calibration curves were constructed using the Bootstrap method with 1,000 resamples, showing good agreement between predicted probabilities and observed event rates; the calibration curves were close to the ideal reference line, indicating accurate and reliable model predictions (Figure 7A–C).

**FIGURE 8:**
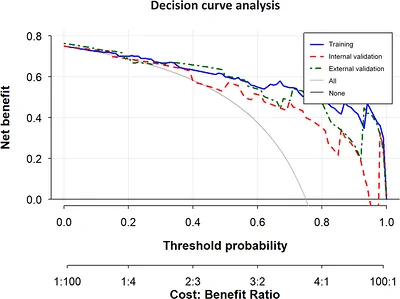
DCA of the training set, internal validation set, and external validation set. DCA, decision curve analysis.

DCA demonstrated that the model provided positive net clinical benefit within a threshold probability range of approximately 0.05–0.9 (corresponding to a cost‐benefit ratio of −1:20 to −20:1) in both the training and validation sets (Figure 8). Within this range, using the model for clinical decision‐making (e.g., stratifying patients by PSD risk) yielded greater net benefit than treating all patients or none. However, we acknowledge that the model's clinical utility is context‐dependent: the threshold probability must align with clinical priorities (e.g., resource allocation, acceptable risk tolerance) to realize this benefit. In summary, this model not only exhibits good discriminative power and calibration accuracy but also demonstrates conditional practical value for clinical decision‐making, provided the threshold probability falls within the identified range.

### 3.5 Sensitivity Analysis

To evaluate whether the predictive value of the three fNIRS features is independent of conventional clinical variables, we performed an additional multivariable logistic regression analysis in the training set. The model included the three selected fNIRS features together with all available clinical variables: age, sex, hypertension, diabetes, stroke cause, lesion site, smoking, drinking, and heart disease. All three fNIRS features remained statistically significant independent predictors of severe PSD after standardization (LIPG_RIPG: OR = 32.28, 95% CI: 4.11–549.19, *p* = 0.005; RDLPFC_RVAC: OR = 22.25, 95% CI: 4.36–227.88, *p* = 0.002; LFEF_LFEF: OR = 19.57, 95% CI: 3.95–195.35, *p* = 0.002). In contrast, none of the clinical variables reached statistical significance (all *p* > 0.10). These results confirm that the identified fNIRS connectivity features provide independent predictive value beyond conventional clinical factors, supporting the robustness of our main findings. (See Table S2 for full results.)

To assess whether the association between fNIRS features and PSD severity is dependent on dichotomization, we performed a linear regression analysis using the original continuous SSA score (range 18–46) as the outcome, adjusting for all clinical covariates (age, sex, hypertension, diabetes, stroke cause, lesion site, smoking, drinking, and heart disease). As shown in Table S3, all three fNIRS features remained statistically significant independent predictors of higher SSA scores (LIPG_RIPG: *β* = 1.22, 95% CI: 0.14–2.31, *p* = 0.030; RDLPFC_RVAC: *β* = 1.19, 95% CI: 0.11–2.27, *p* = 0.032; LFEF_LFEF: *β* = 1.13, 95% CI: 0.11–2.15, *p* = 0.033). None of the clinical variables reached significance (all *p* > 0.05). These results confirm that the predictive value of the fNIRS features is robust and not an artifact of the chosen dichotomization threshold.

Sensitivity analysis utilizing inside‐loop SMOTE and ROSE resampling demonstrated that the overall discriminative capacity of the predictors remained highly resilient, with the cross‐validated AUC consistently staying above 0.90 across all balancing scenarios (Original vs. SMOTE vs. ROSE: 0.906 vs. 0.910 vs. 0.909). Although data balancing successfully rectified the classification boundary by increasing the specificity (up to 0.888), it led to an unacceptable drop in sensitivity (down to 0.757). Detailed evaluation metrics are reported in Figure S3.

## 4 Discussion

This study successfully developed and externally validated a nomogram model based on resting‐state fNIRS–derived brain functional connectivity to predict the severity of PSD. The final model incorporated three robust connectivity indicators—LIPG_RIPG, RDLPFC_RVAC, and LFEF_LFEF—and demonstrated excellent discrimination, with AUCs of 0.928 (training set), 0.857 (internal validation set), and 0.881 (in the external validation set), alongside sound calibration and favorable clinical net benefit. These findings highlight the model's reliability and translational potential. Despite advances in stroke care, the neurophysiological mechanisms and multifactorial determinants of PSD remain incompletely understood, and clinically practical, cost‐effective tools for early risk stratification are still lacking. By integrating objective neuroimaging biomarkers, the present model addresses this gap, offering a non‑invasive, easily implementable instrument for individualized PSD risk assessment. Beyond risk prediction, its outputs may guide tailored rehabilitation planning in clinical and nursing practice, thereby contributing to more proactive and precise PSD management.

From the perspective of neurophysiological mechanisms and anatomical basis, the three functional connectivity predictor variables selected in this study are all HbO‐related features. This aligns closely with the neurovascular coupling principle underlying fNIRS, as HbO serves as a core indicator more sensitive to cortical activation, and its concentration changes can effectively reflect neural activity (Lin et al. 2023; Shah and Seghouane 2014; Tachtsidis and Scholkmann 2016). These features carry clear neurofunctional significance. Firstly, LIPG_RIPG represents a crucial component of the swallowing network. As a key region involved in swallowing motor planning, the bilateral coordinated activity of the IPG is essential for the initiation and execution of swallowing. Our finding of reduced connectivity in this pathway could be related to impaired inter‐hemispheric coordination, which is consistent with the hypothesis that recovery of swallowing function after stroke relies on bilateral compensatory synergy (Inamoto et al. 2015; Ma et al. 2024; Wang et al. 2024). Future direct experimental evidence is still needed to validate this speculation. Secondly, RDLPFC_RVAC implicates the role of higher‐order cognition and sensory integration in swallowing. The DLPFC is involved in attentional allocation and motor decision‐making, while the VAC contributes to visually guided swallowing coordination. The observed aberrant connectivity between these regions might indicate disruption of supramodal regulation or multisensory integration (Cheng et al. 2022; Inamoto et al. 2015; Kang et al. 2024; H. Liu et al. 2022; Ma et al. 2024; Wang et al. 2024; Yuan et al. 2015). Finally, LFEF_LFEF serves as an indicator of local brain regional synchrony. The FEF participate in the spatial localization and initiation of swallowing actions through interactions with the frontoparietal attention network. Our results suggest that functional abnormalities in this region may be associated with decreased attention to swallowing‐related sensory stimuli, which could theoretically reduce swallowing efficiency. Nevertheless, this mechanism remains hypothetical and awaits direct testing, but alternative explanations cannot be ruled out without interventional or lesion‐based studies (Cheng et al. 2022; Duverne and Koechlin 2017; Ma et al. 2024; Yuan et al. 2015). Collectively, these findings are consistent with the idea that swallowing depends on coordinated regulation across multiple brain regions and that altered functional connectivity may contribute to PSD severity. However, correlational fNIRS data cannot establish causality, and further research using multimodal approaches (e.g., transcranial magnetic stimulation, lesion‐symptom mapping) is needed to validate the proposed neural mechanisms.

A diverse array of clinical tools is currently available for the assessment of PSD, including the SSA, the Kawasaki Water Swallowing Test (KWST) (Boaden et al. 2021), the Volume‐Viscosity Swallow Test (V‐VST) (Z. Liu et al. 2020; T. Ye et al. 2018), the Functional Oral Intake Scale (FOIS) (Galovic et al. 2019), the National Institutes of Health Stroke Scale (NIHSS) (Alemseged et al. 2022), and videofluoroscopic swallowing study (VFSS), which is regarded as the gold standard (Na et al. 2019). Among these, scale‐based tools are characterized by their convenience and ease of bedside implementation; however, their results are often influenced by the operator's subjective experience and lack support from objective, quantifiable indicators. Although the NIHSS is widely used to assess stroke severity and prognosis, it comprises numerous items, making its administration relatively cumbersome during the acute phase. Moreover, as a comprehensive neurological assessment instrument, it is not specific to swallowing function and is therefore insufficient for accurately predicting PSD—a complex outcome influenced by multiple factors (Alemseged et al. 2022). VFSS, while capable of providing detailed physiological information about the swallowing process, is limited by its operational complexity and high cost, rendering it impractical for frequent, large‐scale application in all acute‐phase patients (Marin et al. 2023; Na et al. 2019). Consequently, although several studies have attempted to construct nomograms, machine learning models, or Bayesian networks using clinical features to integrate multiple variables into quantifiable risk scores—thereby offering intuitive and individualized risk assessment tools (Liang et al. 2025; Lu et al. 2025; Seo et al. 2025; F. Ye et al. 2024)—most of these models have failed to incorporate objective and detailed neurophysiological or neuroimaging data (e.g., VFSS, motor evoked potentials [MEP], surface electromyography [sEMG], fNIRS, and functional magnetic resonance imaging [fMRI]). Furthermore, they have not deeply explored the underlying brain functional mechanisms. These limitations restrict their applicability in early precise assessment.

The fNIRS‐based brain functional connectivity model developed in this study offers distinct strengths in predictive performance, methodological rigor, and clinical applicability. With AUCs of 0.928 and 0.857 in the training and internal validation sets, respectively, and robust external performance confirmed in an independent cohort from Sichuan Gem Flower Hospital, the model's discriminative ability surpasses most conventional clinical‑indicator–based approaches and is comparable to several high‑quality PSD prediction models (Lu et al. 2025; F. Ye et al. 2024). Comprehensive validation through calibration curves and DCA further substantiates its accuracy and clinical utility.

Methodologically, we adopted a dual‑stage feature selection strategy combining LASSO regression with bootstrap stability resampling (*B* = 200), effectively mitigating overfitting and multicollinearity while enhancing model robustness. By constructing the model upon whole‑network functional connectivity across multiple swallowing‑related regions, we more comprehensively captured the cooperative neural mechanisms underlying deglutition (Kang et al. 2024; H. Liu et al. 2022; Wang et al. 2024).

Technically, although MRI provides superior spatial resolution and access to deep structures (Flowers et al. 2017; Johansen‑Berg and Rushworth 2009; Nakamori et al. 2025), fNIRS remains inherently limited in detection depth and coverage. Nevertheless, its portability, high tolerance to motion artifacts, and low operational cost make it uniquely suited for bedside, dynamic assessments in acute stroke care (Rieke et al. 2020; Y. Zhao et al. 2024).

Clinically, the model is operationalized as an intuitive nomogram, translating complex brain network data into a straightforward scoring tool. This design minimizes computational barriers, enabling rapid, individualized PSD risk estimation without specialized software, thereby facilitating early identification of high‐risk patients and guiding precise interventions. Beyond prediction, the identified connectivity features yield neurophysiological insights into PSD mechanisms and delineate potential neuromodulatory targets, extending the model's value from risk stratification to mechanistic exploration and personalized rehabilitation planning.

It should be noted that the ratio of mild to severe PSD cases in the training set was approximately 1:3, reflecting a moderate degree of class imbalance. To address this, we applied the SMOTE and ROSE algorithms to resample the training set to a 1:1 ratio and refitted the logistic regression models using the same set of predictors. The results showed that the balanced models achieved an AUC comparable to that of the original model; however, their sensitivity decreased. Given that the core goal of clinical screening is to identify severe PSD patients as early as possible to avoid serious complications such as aspiration and pneumonia, high sensitivity is more clinically valuable than high specificity. Consequently, the original imbalanced model better aligns with real‐world clinical priorities, and its overall discriminative ability was not substantially compromised by the class imbalance. Nevertheless, the model's performance requires further confirmation in larger, independent validation sets.

Several limitations of this study should be addressed in future research. First, the internal validation set included only 40 patients, with only 10 mild PSD cases, which may affect the stability of performance estimates and widen the CI. Although we have supplemented the analysis with a small‑sample independent external validation, larger multicenter prospective studies are still needed to comprehensively evaluate the generalizability of the model. Second, at the data level, fNIRS technology itself has inherent limitations, including limited detection depth and susceptibility to physiological noise, which preclude direct monitoring of deep swallowing nuclei such as those in the brainstem. Moreover, only resting‐state fNIRS signals were acquired in this study; task‐state swallowing data were not collected, potentially failing to fully capture swallowing‐specific brain functional responses. Regarding model construction, to maintain simplicity and clinical usability, only three fNIRS‐derived functional connectivity features were ultimately included. Although this model achieved satisfactory performance, other potentially valuable clinical (e.g., rehabilitation interventions) or imaging predictors may not have been incorporated, which could limit its comprehensive predictive power. Furthermore, the cross‐sectional design only established statistical associations between brain functional characteristics and swallowing scores, precluding inferences of causality. It also did not evaluate the model's predictive value for long‐term functional recovery or other clinical outcomes. Future research should integrate multimodal imaging and longitudinal follow‐up data to validate and optimize the model's clinical applicability in broader populations. Additionally, combining this approach with neuromodulation techniques may enable the development of individualized rehabilitation strategies targeting key brain regions identified by the model, ultimately improving patient prognosis.

## 5 Conclusion

This study successfully developed and externally validated a nomogram model based on resting‐state fNIRS–derived brain functional connectivity to predict the severity of PSD. The final model incorporated three robust predictors—LIPG_RIPG, RDLPFC_RVAC, and LFEF_LFEF—representing key connections within the swallowing‑related network and demonstrated excellent discrimination, calibration, and clinical net benefit, with stable performance confirmed in an external validation set. These findings underscore the value of fNIRS as an objective, non‑invasive, and clinically feasible neuroimaging modality for elucidating the neural mechanisms underlying PSD and enabling precise, individualized risk assessment. The resulting nomogram translates complex brain network data into an intuitive, quantifiable tool that allows clinicians to rapidly identify high‑risk patients and to inform neuroscience‐guided, personalized rehabilitation strategies. Beyond risk stratification, the model highlights candidate neural targets for future neuromodulatory interventions, thereby extending its impact from acute clinical decision‑making to long‑term functional recovery and optimized patient outcomes.

## Author Contributions

**Yizheng Li**: investigation, validation. **Rong Zhang**: conceptualization, methodology, investigation, formal analysis. **Yulei Xie**: methodology, supervision, resources, project administration, writing – review and editing, funding acquisition, formal analysis. **Qian Wen**: conceptualization, methodology, data curation, investigation, formal analysis, validation. **Xiaojuan Chen**: data curation, supervision, resources, software. **Yiya Wang**: data curation, investigation, formal analysis, validation. **Bangqiang Hou**: data curation, writing – original draft, visualization, software, validation. **Yinxu Wang**: methodology, data curation, supervision, resources.

## Ethics Statement

The study protocol was reviewed and approved by the Medical Ethics Committee of the Affiliated Hospital of North Sichuan Medical College (Approval No. 2026ER205‐1) and the Medical Ethics Committee of Sichuan Gem Flower Hospital (Approval No. SCBSH‐EC‐26‐23). All procedures were conducted in strict accordance with the ethical principles of the Declaration of Helsinki. Given the retrospective design of this study, the ethics committee granted a waiver for obtaining informed consent.

## Funding

This study was supported by North Sichuan Medical College, Clinical Medical School & Affiliated Hospital, 2025 High‐level Talent Recruitment Scientific Research Start‐up Fund Project (Grant No. 2025GC016); Special Scientific Research Project on Chronic Diseases (Tige) of the Sichuan Medical Association (Grant No. 2025TG04); and Nanchong Social Science Research “15th Five‐Year Plan” 2026 Annual Project (Grant No. NC26C418, NC26C011); Sichuan Provincial Nursing Research Project Program (Grant No. H23046); Sichuan Provincial Health Commission Science and Technology Project (Grant No. 24WSXT086).

## Conflicts of Interest

All authors declare no conflicts of interest.

## Supporting information




**Supplementary Material**: brb371791‐sup‐0001‐SuppMat.docx

## Data Availability

Data supporting the findings of this study are available from the corresponding author upon request.
