# Supplementary material for: Development and Validation of a Nomogram Model for Predicting the Severity of Post‐Stroke Dysphagia Based on fNIRS‐Derived Brain Functional Connectivity Features
Source: Brain Behav. 2026 Sep 23;16(9):e71791. doi: 10.1002/brb3.71791 (PMC13601745; doi:10.1002/brb3.71791)
Supplement: Supplementary file 1 [file BRB3-16-e71791-s001.docx]

**Supplementary Materials**

## **Table S1. Baseline characteristics of the 38 PSD patients.**

| **Variables** | **Total**  **(n = 38)** | **MPSDG**  **(n = 9)** | **SPSDG**  **(n = 29)** | **Statistics** | **p** |
| --- | --- | --- | --- | --- | --- |
| **Age**, (years), (median, IQR) | 68.00 (59.75,74.00) | 68.00 (53.50, 80.50) | 68.00 (60.50, 74.00) | 121.500^a^ | 0.768 |
| **Sex**, n (%) |  |  |  | 1.083^b^ | 0.298 |
| Male | 24 (63.2) | 7 (77.8) | 17 (58.6) |  |  |
| Female | 14 (36.8) | 2 (22.2) | 12 (41.4) |  |  |
| **Stroke types**, n (%) |  |  |  | 0.102^b^ | 0.750 |
| Ischemic | 28 (73.7) | 7 (77.8) | 21 (72.4) |  |  |
| Hemorrhagic | 10(26.3) | 2 (22.2) | 8 (27.6) |  |  |
| **Site of stroke**, n (%) |  |  |  | 0.004^b^ | 0.949 |
| Left | 13 (34.2) | 3 (33.3) | 10 (34.5) |  |  |
| Right | 25 (65.8) | 6 (66.7) | 19 (65.5) |  |  |
| **Hypertension**, n (%) |  |  |  | 0.903^b^ | 0.342 |
| No | 12 (31.6) | 4 (44.4) | 8 (27.6) |  |  |
| Yes | 26 (68.4) | 5 (55.6) | 21 (72.4) |  |  |
| **Heart disease**, n (%) |  |  |  | 0.102^b^ | 0.750 |
| No | 28 (73.7) | 7 (77.8) | 21 (72.4) |  |  |
| Yes | 10 (26.3) | 2 (22.2) | 8 (27.6) |  |  |
| **Diabete**s, n (%) |  |  |  | 2.918^b^ | 0.088 |
| No | 22 (57.9) | 3 (33.3) | 19 (65.5) |  |  |
| Yes | 16 (42.1) | 6 (66.7) | 10 (34.5) |  |  |
| **Smoking**, n (%) |  |  |  | 0.620^b^ | 0.431 |
| No | 21 (55.3) | 6 (66.7) | 15 (51.7) |  |  |
| Yes | 17 (44.7) | 3 (33.3) | 14 (48.3) |  |  |
| **Drinking**, n (%) |  |  |  | 0.186^b^ | 0.666 |
| No | 23 (60.5) | 6 (66.7) | 17 (58.6) |  |  |
| Yes | 15 (39.5) | 3 (33.3) | 12 (41.4) |  |  |
| **SSA**, (median, IQR) | 28.00 (25.75, 32.00) | 23.00 (21.50, 23.00) | 30.00 (27.50, 32.00) |  |  |

Abbreviations: MPSDG, mild PSD group; SPSDG, severe PSD group; SSA, standardized swallowing sssessment.^a^ Using the Mann -Whitney U test; ^b^ Using the χ² test.

**Table S2. Multivariable logistic regression analysis adjusting for clinical covariates.**

| **Variable** | **OR (95% CI)** | **p value** |
| --- | --- | --- |
| **LIPG_RIPG** | 32.28 (4.11–549.19) | 0.005 |
| **RDLPFC_RVAC** | 22.25 (4.36–227.88) | 0.002 |
| **LFEF_LFEF** | 19.57 (3.95–195.35) | 0.002 |
| **Age** | 1.05 (0.97–1.14) | 0.252 |
| **Sex** | 0.52 (0.06–3.62) | 0.513 |
| **Hypertension** | 0.17 (0.01–1.33) | 0.118 |
| **Diabetes** | 0.42 (0.07–2.3) | 0.327 |
| **Causes** | 0.66 (0.09–5.16) | 0.675 |
| **Site_of_injury** | 1.07 (0.22–5.51) | 0.931 |
| **Smoking** | 4.09 (0.23–90.44) | 0.342 |
| **Drinking** | 1.56 (0.09–32.6) | 0.761 |
| **Heart_disease** | 0.36 (0.05–2.31) | 0.302 |

The model was fitted in the training set (n = 100) with severe PSD as the outcome. All three fNIRS features were standardised (Z‑score) before entry; thus, the odds ratios (ORs) represent the change in odds of severe PSD per one standard deviation increase in the original connectivity strength. The model also included all available clinical variables. Data are presented as OR with 95% confidence interval and p‑value. The wide CIs for the fNIRS features reflect the relatively small sample size, but all lower bounds are >1 and p‑values <0.01, confirming statistical significance. None of the clinical variables reached significance (all p > 0.10). Abbreviations: CI, confidence interval; RDLPFC, right dorsolateral prefrontal cortex; left frontal eye fields; L/RIPG, left/right inferior prefrontal gyrus; LFEF, left frontal eye fields; RVAC, right visual association cortex; LIPG_RIPG, functional connectivity between the LIPG and RIPG; RDLPFC_RVAC, functional connectivity between the RDLPFC and RVAC; LFEF_LFEF, intra-regional functional connectivity within the LFEF.

**Table S3: Sensitivity analysis using continuous SSA score (linear regression).**

| **Variables** | **β (95% CI)** | **P_value** |
| --- | --- | --- |
| **LIPG_RIPG** | 1.22 (0.14, 2.31) | 0.03 |
| **RDLPFC_RVAC** | 1.19 (0.12, 2.27) | 0.032 |
| **LFEF_LFEF** | 1.13 (0.11, 2.15) | 0.033 |
| **Age** | 0.04 (-0.04, 0.13) | 0.318 |
| **Sex** | 0.08 (-2.37, 2.53) | 0.952 |
| **Hypertension** | 0.29 (-1.81, 2.39) | 0.788 |
| **Diabetes** | -1.44 (-3.55, 0.66) | 0.183 |
| **Stroke causes** | 0.34 (-1.99, 2.66) | 0.777 |
| **Lesion site** | -1.02 (-3, 0.96) | 0.316 |
| **Smoking** | -0.9 (-4.1, 2.3) | 0.582 |
| **Drinking** | 1.07 (-1.93, 4.06) | 0.487 |
| **Heart disease** | -0.53 (-3.24, 2.19) | 0.705 |

All fNIRS features were Z‑score standardised; β represents the average increase in SSA score (range 18–46) per one standard deviation increase in the original connectivity strength. The model was adjusted for age, sex, hypertension, diabetes, stroke cause, lesion site, smoking, drinking, and heart disease. Data are β (95% CI) and p‑value. Abbreviations: SSA, Standardized Swallowing Assessment; CI, confidence interval; RDLPFC, right dorsolateral prefrontal cortex; left frontal eye fields; L/RIPG, left/right inferior prefrontal gyrus; LFEF, left frontal eye fields; RVAC, right visual association cortex; LIPG_RIPG, functional connectivity between the LIPG and RIPG; RDLPFC_RVAC, functional connectivity between the RDLPFC and RVAC; LFEF_LFEF, intra-regional functional connectivity within the LFEF.

**Figure S1.** **Distribution of model performance metrics under repeated 5‑fold cross‑validation.** Red diamonds represent the mean value of each metric across the 25 cross‑validation iterations; colored scatter points show the results of individual folds (N = 25). The boxplots illustrate the distribution characteristics: the black horizontal line inside each box denotes the median, the upper and lower bounds of the box correspond to the upper and lower quartiles, respectively, and the whiskers extend to the most extreme values within 1.5 times the interquartile range. The evaluated metrics include AUC, sensitivity, specificity, balanced accuracy, and F1 score for severe outcome.

**Figure S2. Summary of mean and standard deviation of cross‑validation performance.** The bar height represents the mean score of each evaluation metric across the repeated 5‑fold cross‑validation, and the error bars indicate the corresponding standard deviation, reflecting the degree of fluctuation in model performance. *Abbreviations:* AUC, area under the curve.

**Figure S3. Sensitivity analysis of model performance under different class-balancing techniques.** The original imbalanced model achieved the highest sensitivity (0.917), while AUC values remained comparable across all three models (0.906–0.910). Balancing increased specificity but substantially reduced sensitivity. Based on the clinical need to prioritize detection of severe PSD, the original model was retained. *Abbreviations:* AUC, area under the curve.
